# Supplementary material for: Prevalence of mental health conditions in post-conflict Kasai Province, Democratic Republic of the Congo: A repeated, cross-sectional study
Source: PLOS Glob Public Health. 2025 Jan 9;5(1):e0004057. doi: 10.1371/journal.pgph.0004057 (PMC11717290; doi:10.1371/journal.pgph.0004057)
Supplement: S1 Appendix — (DOCX) [file pgph.0004057.s001.docx]

S1 Appendix: Mental health outcomes across wave and sex

|  | **Wave 1** | **Wave 2** |  | **Wave 1** | **Wave 2** |  | **Wave 1** | **Wave 2** |  | **Wave 1** | |  | **Wave 2** | |  |
| --- | --- | --- | --- | --- | --- | --- | --- | --- | --- | --- | --- | --- | --- | --- | --- |
|  | Overall | Overall |  | Men | Men |  | Women | Women |  | Men | Women |  | Men | Women |  |
| *Depression* | n=200 | n=185 |  | n=107 | n=96 |  | n=93 | n=89 |  | n=107 | n=93 |  | n=96 | n=89 |  |
| Mean  (SD) | 8.4 (5.6) | 7.4 (4.6) |  | 7.5 (5.2) | 7.0 (4.5) |  | 9.5 (5.8) | 7.8 (4.7) |  | 7.5 (5.2) | 9.5  (5.8) |  | 7.0 (4.5) | 7.8 (4.7) |  |
| Major depression  (PHQ-9≥10) | 71 (35.5) | 61 (33.0) | Z= n.s. | 31 (29.0) | 30 (31.3) | Z=n.s. | 40 (43.0) | 31 (34.8) | Z=n.s. | 31 (29.0) | 40  (43.0) | **Z = -2.06, *p=*0.04** | 30 (31.3) | 31 (34.8) | Z=n.s. |
| None  (0-4) | 56 (28.0) | 58 (31.4) |  | 35 (32.7) | 32 (33.3) |  | 21 (22.6) | 26 (29.2) |  | 35 (32.7) | 21 (22.6) |  | 32 (33.3) | 26 (29.2) |  |
| Mild  (5-9) | 73 (36.5) | 66 (35.7) |  | 41 (38.3) | 34 (35.4) |  | 32 (34.4) | 32 (36.0) |  | 41 (38.3) | 32 (34.4) |  | 34 (35.4) | 32 (36.0) |  |
| Moderate  (10-14) | 35 (17.5) | 48 (25.9) |  | 16 (15.0) | 25 (26.0) |  | 19 (20.4) | 23 (25.8) |  | 16 (15.0) | 19 (20.4) |  | 25 (26.0) | 23 (25.8) |  |
| Mod.-severe  (15-19) | 29 (14.5) | 11  (5.9) |  | 13 (12.1) | 5  (5.2) |  | 16 (17.2) | 6  (6.7) |  | 13 (12.1) | 16 (17.2) |  | 5 (5.2) | 6  (6.7) |  |
| Severe  (20-27) | 7  (3.5) | 2  (1.1) |  | 2  (1.9) | 0  (0.0) |  | 5  (5.4) | 2  (2.2) |  | 2 (1.9) | 5  (5.4) |  | 0 (0.0) | 2  (2.2) |  |
|  |  |  |  |  |  |  |  |  |  |  |  |  |  |  |  |
| *Suicidal/self-harm* | 48 (24.0) | 27 (14.6) | **Z=2.33, *p=*0.02** | 19 (17.8) | 10 (10.4) | Z=n.s. | 29 (31.2) | 17 (19.1) | Z= n.s. | 19 (17.8) | 29  (31.2) | **Z= -2.22, *p=*0.03** | 10 (10.4) | 17 (19.1) | Z=n.s. |
|  |  |  |  |  |  |  |  |  |  |  |  |  |  |  |  |
| *Anxiety* |  |  |  |  |  |  |  |  |  |  |  |  |  |  |  |
| Mean  (SD) | 7.2 (4.6) | 6.6 (3.9) |  | 7.0 (4.4) | 6.6 (3.9) |  | 7.3 (4.7) | 6.6 (3.9) |  | 7.0 (4.4) | 7.3  (4.7) |  | 6.6 (3.9) | 6.6 (3.9) |  |
| Anxiety  (GAD-7≥10) | 62 (31.0) | 40 (21.6) | **Z =2.08, *p*=0.04** | 30 (28.0) | 23 (24.0) | Z=n.s. | 32 (34.4) | 17 (19.1) | **Z = -2.33, *p=*0.02** | 30 (28.0) | 32  (34.4) | Z= n.s. | 23 (24.0) | 17 (19.1) | Z=n.s. |
| None  (0-4) | 70 (35.0) | 61 (33.0) |  | 36 (33.6) | 32 (33.3) |  | 34 (36.6) | 29 (32.6) |  | 36 (33.6) | 34 (36.6) |  | 32 (33.3) | 29 (32.6) |  |
| Mild  (5-9) | 68 (34.0) | 84 (45.4) |  | 41 (38.3) | 41 (42.7) |  | 27 (29.0) | 84 (45.4) |  | 41 (38.3) | 27 (29.0) |  | 41 (42.7) | 84 (45.4) |  |
| Moderate  (10-14) | 50 (25.0) | 34 (18.4) |  | 24 (22.4) | 21 (21.9) |  | 26 (28.0) | 13 (14.6) |  | 24 (22.4) | 26 (28.0) |  | 21 (21.9) | 13 (14.6) |  |
| Severe  (>15) | 12  (6.0) | 6  (3.2) |  | 6  (5.6) | 2  (2.1) |  | 6  (6.5) | 4  (4.5) |  | 6 (5.6) | 6  (6.5) |  | 2 (2.1) | 4  (4.5) |  |
