# Supplementary material for: Prevalence of mental health conditions in post-conflict Kasai Province, Democratic Republic of the Congo: A repeated, cross-sectional study
Source: PLOS Glob Public Health. 2025 Jan 9;5(1):e0004057. doi: 10.1371/journal.pgph.0004057 (PMC11717290; doi:10.1371/journal.pgph.0004057)
Supplement: S2 Appendix — (DOCX) [file pgph.0004057.s002.docx]

S2 Appendix QoL mean-per-item scores, per sex

|  | **Wave 1** | | | **Wave 2** | | |
| --- | --- | --- | --- | --- | --- | --- |
|  | Overall | Men | Women | Overall | Men | Women |
| **Item** | M (SD) | M (SD) | M (SD) | M (SD) | M (SD) | M (SD) |
| 1. Overall QoL | 2.8 (1.2) | 2.5 (1.2) | 3.2 (1.0) | 3.0 (1.2) | 2.8 (1.2) | 3.3 (1.0) |
| 1. General Health | 3.2 (1.2) | 3.1 (1.3) | 3.3 (1.1) | 3.2 (1.3) | 3.3 (1.3) | 3.4 (1.1) |
| 1. Physical Health |  |  |  |  |  |  |
| 3. Pain and discomfort | **3.1 (1.6)** | 3.1 (1.5) | 3.1 (1.6) | **3.4 (1.4)*** | 3.3 (1.3) | 3.5 (1.6) |
| 4. Dependence on medication | 2.0 (1.4) | 1.8 (1.2) | 2.3 (1.5) | 1.9 (1.3) | 2.0 (1.1) | 1.9 (1.5) |
| 10. Energy | Missing | Missing | Missing | 3.6 (1.4) | 3.3 (1.3) | 4.0 (1.4) |
| 15. Mobility | 2.9 (1.2) | 2.7 (1.2) | 3.1 (1.1) | 2.7 (1.2) | 2.8 (1.2) | 2.6 (1.1) |
| 16. Sleep | **3.2 (1.2)** | 3.0 (1.3) | 3.4 (1.0) | **3.5 (1.1)*** | 3.4 (1.2) | 3.6 (1.0) |
| 17. Activities of daily living | 3.6 (1.1) | 3.6 (1.0) | 3.6 (0.9) | 3.7 (1.0) | 3.5 (1.1) | 3.9 (0.6) |
| 18. Work capacity | **3.7 (0.9)** | 3.7 (1.0) | 3.7 (0.8) | **3.9 (0.8)*** | 3.9 (0.9) | 3.9 (0.5) |
| 1. Psychological health |  |  |  |  |  |  |
| 5. Enjoying life | 3.1 (1.6) | 2.9 (1.6) | 3.4 (1.5) | 3.0 (1.7) | 2.6 (1.4) | 3.5 (1.7) |
| 6. Meaningful life | **3.7 (1.7)** | 3.8 (1.2) | 3.6 (1.3) | **4.1 (1.1)*** | 4.1 (0.9) | 4.1 (1.4) |
| 7. Concentration | 3.5 (1.4) | **3.6 (1.3)** | 3.3 (1.6) | 3.5 (1.4) | **3.3 (1.2)*** | 3.8 (1.5) |
| 11. Body image | 4.1 (1.4) | 4.3 (1.1) | 3.8 (1.6) | 4.2 (1.3) | 4.0 (1.1) | 4.4 (1.3) |
| 19. Self-esteem | 3.2 (1.5) | 3.4 (1.4) | 3.0 (1.5) | 3.2 (1.5) | 3.2 (1.3) | 3.1 (1.7) |
| 26. Negative feelings | 4.3 (1.3) | 4.4 (1.2) | 4.2 (1.3) | 4.5 (1.1) | 4.7 (0.9) | 4.2 (1.3) |
| 1. Social relationships |  |  |  |  |  |  |
| 20. Personal relations | **3.1 (1.3)** | 2.9 (1.3) | 3.4 (1.0) | **2.8 (1.3)*** | 2.7 (1.3) | 2.9 (1.3) |
| 21. Sex | 3.9 (0.9) | 4.1 (0.7) | 3.6 (0.9) | 4.0 (1.0) | 4.3 (0.9) | 3.6 (1.0) |
| 22. Social support | 3.9 (0.9) | 3.9 (0.9) | 3.8 (0.7) | 3.9 (0.9) | 3.9 (1.0) | 3.9 (0.8) |
| 1. Environment |  |  |  |  |  |  |
| 8. Safety | 3.3 (1.5) | 3.2 (1.5) | **3.3 (1.5)** | 3.5 (1.4) | 3.4 (1.3) | **3.7 (1.5)*** |
| 9. Physical environment | **3.2 (1.4)** | 2.9 (1.4) | 3.5 (1.3) | **3.5 (1.3)*** | 3.6 (1.1) | 3.4 (1.4) |
| 12. Finances | 1.3 (0.9) | 1.2 (0.8) | 1.4 (1.0) | 1.2 (0.6) | 1.2 (0.6) | 1.2 (0.6) |
| 13. Information | 4.0 (1.5) | 4.1 (1.3) | 3.8 (1.6) | 3.7 (1.4) | 3.5 (1.3) | 3.9 (1.4) |
| 14. Leisure | 2.4 (1.6) | 2.3 (1.6) | 2.4 (1.7) | 2.3 (1.6) | 2.4 (1.5) | 2.2 (1.7) |
| 23. Living conditions | 3.7 (1.0) | **3.5 (1.3)** | 3.8 (0.7) | 3.7 (1.0) | **3.8 (1.0)*** | 3.5 (1.0) |
| 24. Access to healthcare | 2.4 (1.7) | 2.1 (1.6) | 2.7 (1.7) | 2.3 (1.5) | 2.1 (1.3) | 2.5 (1.6) |
| 25. Transport | 2.5 (1.4) | 2.4 (1.5) | 2.7 (1.3) | 2.4 (1.3) | 1.8 (1.1) | 3.0 (1.2) |
