# Supplementary material for: Prevalence of mental health conditions in post-conflict Kasai Province, Democratic Republic of the Congo: A repeated, cross-sectional study
Source: PLOS Glob Public Health. 2025 Jan 9;5(1):e0004057. doi: 10.1371/journal.pgph.0004057 (PMC11717290; doi:10.1371/journal.pgph.0004057)
Supplement: Attachment — Submitted filename: DRC_Prevalence of mental health conditions in post.docx [file pgph.0004057.s006.docx]

Prevalence of mental health conditions in post-conflict Kasai Province, Democratic Republic of the Congo: a repeated, cross-sectional study.

Thank you for the opportunity to review the paper.

Here are my observations:

1.**This is an important study.**

**2.The repeat survey at one year interval is its strength.**

**3.The study provides insights tio plan services and multi dimensional interventions.**

**4.Discussion could include more information about the qualitative aspects of life for the population in general and women in particular.**

**5.The area ofor inclusion is the envcpouragewnent of SELF CARE For EMOTIONAL HEALTH. This is an emerging area in disaster mental health.**

My recommendation is Accept the manuscript, with additions,  for publication.

R.Srinivasa Murthy

Bangalore
