# Supplementary material for: Prevalence of mental health conditions in post-conflict Kasai Province, Democratic Republic of the Congo: A repeated, cross-sectional study
Source: PLOS Glob Public Health. 2025 Jan 9;5(1):e0004057. doi: 10.1371/journal.pgph.0004057 (PMC11717290; doi:10.1371/journal.pgph.0004057)
Supplement: Attachment — Submitted filename: Rebuttal letter3_14.10.2024.docx [file pgph.0004057.s009.docx]

REBUTTAL LETTER: “Prevalence of mental health conditions in post-conflict Kasai Province, Democratic Republic of the Congo: a repeated, cross-sectional study”

We thank the reviewers for taking the time to review our paper and for the useful feedback provided. Please see below for our point-by-point responses. We hope we have addressed each comment satisfactorily and that the paper is now suited for publication.

Reviewer #1: (No Response)

Reviewer #3: Tables should converted to Scientific tables (Editor will decide) We can confirm tables are in the correct format.
Line 133: Statement “Questionnaires were translated and back-translated from English into French”. Why was it translated in English? It was translated in English, because the that was the working language of the partnership team. In addition, the study tools that we used we originally developed in English. The tools were then translated in French, because this is the language used by the in-country teams.
Line 160: Table 1 Demographic characteristics of study participants should be “Table 1: Demographic characteristics of study participants”. Thank you, this has now been changed.
Table 1: Age should have a unit i.e. Age (years). This has now been added.
Line 161: average 40.0 years old (SD = 15.0) should be written as “40.0±15.0 years”. This has now been changed.
Table 2: Age should have a unit i.e. Age (years). This has been added
Table 4: Age should have a unit i.e. Age (years). This has been added.
Line 205: Discussion – to be consistent, the Percentages be reported to 1 decimal place i.e. 34.0% and not 34%. Decimal places have now been added.

Reviewer #5: None.
Congratulations upon a well written manuscript.

Reviewer #6: This study is a repeated cross-sectional study that investigated the prevalence of depression and anxiety in a post-conflict population in Kasai Province, DRC. This is important because the findings could guide data-informed decision making for the funding and commissioning of mental health services within DRC.

I also agree that the authors' responses to the reviewers' comments were thorough and well-considered. Using "condition" instead of "disease" is appropriate given that formal diagnoses were not made, and the minor points you raise are valid and should be addressed as follows:

a) Line 120: The sentence "...School of Tropical Medicine (reference number: (21-053)." is missing a closing parenthesis. Please add a closing parenthesis after "21-053". Alternatively, you could rewrite the phrase as "(reference number: 21-053)." Thank you for spotting this. This has now been changed.

b) The journal name in reference 14 should be "JAMA", not "Jama". This has been changed.
