# Supplementary material for: Prevalence of mental health conditions in post-conflict Kasai Province, Democratic Republic of the Congo: A repeated, cross-sectional study
Source: PLOS Glob Public Health. 2025 Jan 9;5(1):e0004057. doi: 10.1371/journal.pgph.0004057 (PMC11717290; doi:10.1371/journal.pgph.0004057)
Supplement: Attachment — Submitted filename: Response to Reviewers_MS.docx [file pgph.0004057.s007.docx]

PLOS Global Public Health

2^nd^ May 2023

Dear Sir/Madam,

Re: PGPH-D-23-01634:

Thank you for the timely review of our manuscript. Apologies for the delay in response on our side, as I was on maternity leave. We believe the comments have strengthened our contribution and hope that they adequately address the concerns raised to enable progression to publication. Please find a table in Annex One that addresses each of the concerns raised by the reviewers.

On behalf of the authors,

Yours sincerely,

Dr. Maaike Seekles

Liverpool School of Tropical Medicine

Maaike.seekles@lstmed.ac.uk

***Annex One: Response to Reviewer Comments***

| **Reviewer Comments** | **Response** |
| --- | --- |
| Editor |  |
| Please include a complete copy of PLOS’ questionnaire on inclusivity in global research in your revised manuscript. | This is now included. |
| Please amend your detailed Financial Disclosure statement. This is published with the article. It must therefore be completed in full sentences and contain the exact wording you wish to be published. | This has been updated accordingly. |
| Please provide separate figure files in .tif or .eps format. | These are now provided. |
| We have noticed that you have uploaded Supporting Information files, but you have not included a list of legends. Please add a full list of legends for your Supporting Information files after the references list. | This has been completed. |
| Reviewer One |  |
| No data on conflict related traumatic events are presented. This leaves the reader with two questions: on (i) how these proxy indicators of bio-socioeconomic stressors included should be interpreted: Are they remaining conflict related factors or are they common indicators of poverty that were also present before the conflict? If so, how do they – if at all - interact with conflict related mental health outcomes? And (ii) could the assessment of these stressors possibly be associated with the indicators of mental health (clearly stated by the authors for the psychological domain but not for the other three) so that concerns about safety, economy, access to health and transport are rather manifestations of anxiety and depression than the cause of them? It requires a more rigorous argumentation, addressing these two questions, to present the association between mental health and the WHO-QoL Bref environment domain (applying for women only) as a relevant finding. | Thank you for this critical reflection. The lack of data on conflict related traumatic events has been described as a limitation of this study and our discussion around the rational for our focus on broader mental wellbeing (as opposed to that which is solely trauma related) has been further elaborated in the discussion. Please see lines 223-241.  We have also added additional information to the discussion to clarify/nuance the findings around proxy indicators. It is highly likely that these stressors were already present before the conflict, although the conflict may have exacerbated them. We also agree that the relationship between these stressors may be bidirectional and that this paper can not necessarily attribute causality. The following sentence has been added in response in lines 235-237: ‘*In addition, further (qualitative) research would be needed to explore the causal direction of the relationship between stressors and mental health identified in the current paper and the moderating properties of conflict’.* |
| Secondly, on the information of interventions: It is somewhat diffuse how the findings may contribute to future programming or interventions (multi-sectoral interventions addressing broader social determinants). The paper would gain from more clarity in terms of what contributions the study makes. | Thank you for this feedback. We believe that the findings inform future programming through the provision of data that both emphasises the need for enhanced mental health services in the region and provision of a rationale that this should take a broader determinants-based approach. We have worked to clarify this element of our argument further, specifically, in lines 238-244. Data provided in the appendices could also be used to provide estimates for service commissioning/targeting interventions. |
| There are not many considerations about the ethical implications of undertaking surveys in conflict affected populations. It seems pertinent to describe which information of participation risks was communicated to potential participants, if any participants seemed to have been re-traumatized, if interviewers were capable of handling such situations, and if any referrals to further support or treatment were made. | The methodology now explains that data collectors were trained and that the opportunity for referral was offered through a Congolese clinical psychologist. Please see lines 101-108. |
| The paper states that 1 million people were internally displaced (IDP), but not whether they had returned by 2022 and 2023 and not either what proportion of the study population were IDP. It could be assumed that the environmental domain would be related to IDP in some way. | Thank you. It has been added to the limitations that internal displacement (and PTSD and exposure to violence) were not captured by this study. Please see lines 227-229. This would be an important area for future study. |
| Reviewer Two |  |
| Discussion could include more information about the qualitative aspects of life for the population in general and women in particular. | Thank you for this comment. We have added further clarification within the discussion section of the paper that more work is necessary to understand qualitatively the lived experiences of this population in relation to their mental wellbeing and specifically to understand the causal pathways between stressors and mental health outcomes. Please see lines 238-240. |
| The area for inclusion is the aspects SELF CARE For EMOTIONAL HEALTH. This is an emerging area in disaster mental health. | Thank you. Mention of mental health promotion and self-care programmes have now been added to the discussion. Please see lines 253-254. |
