# Supplementary material for: Prevalence of mental health conditions in post-conflict Kasai Province, Democratic Republic of the Congo: A repeated, cross-sectional study
Source: PLOS Glob Public Health. 2025 Jan 9;5(1):e0004057. doi: 10.1371/journal.pgph.0004057 (PMC11717290; doi:10.1371/journal.pgph.0004057)
Supplement: Attachment — Submitted filename: Rebuttal letter2_09.07.2024.docx [file pgph.0004057.s008.docx]

REBUTTAL LETTER: “Prevalence of mental health conditions in post-conflict Kasai Province, Democratic Republic of the Congo: a repeated, cross-sectional study”

We thank the reviewers for taking the time to review our paper and for the detailed feedback provided. Please see below for our point-by-point responses. We hope we have addressed each comment satisfactorily and feel that it has greatly improved the quality of our paper.

**Reviewer #1:** None

**Reviewer #3:** General comments and some key concerns:
1. It is an interesting study that is giving an insight on the “Prevalence of mental health conditions in post-conflict Kasai Province, Democratic Republic of the Congo: a repeated, cross-sectional study.”

•Suggest “conditions” be replaced with “disorders”. Apply this in the entire document. Thank you for this suggestion. However, within our research group, we prefer to use the term “conditions” over “disorders”, as we feel that this is a less stigmatising term. We would therefore like to request that we leave ‘conditions’ as is.

•Replace “ sample” with “study participants” all through the document. This has been changed where appropriate.

•Avoid long sentences as the meaning get lost – Use short sentences. Sentences have been made shorter where possible.

•Percentages be reported to 1 decimal place. This has been done.

•Tables should converted to Scientific tables. We believe the tables follow the journal guidelines, but am happy to email the journal to confirm and will change if necessary.
•Use term “Study participants” other than “sample” all through the document. This has been changed.

2. Abstract
•Much as it is a block abstract, the following sub-sections should be clear in it (Background, aim, methods, results and conclusion)
•Background information in Mental health disorders in Kasai Province, Democratic Republic of the Congo is missing. There is currently no data available on MH disorders in Kasai. Further background information has been added to the introduction of the overall paper.
•Aim of the study is missing. The word ‘aim’ has now been added to sentence 29.
•Line 34: Percentages be reported to 1 decimal place i.e. 34.0%. APA guidelines suggest that percetnages are rounded up as much as possible while considering prospective use and statistical precision. Decimal places have been added in the results section, to provide greater precision of our data.
•Conclusion is missing. ‘The words ‘in conclusion’ have now been added to sentence 39.
•Use third person in sentence construction – see line 85. We have followed APA guidelines, which recommends referring to yourself in the first person instead of third person.

3. Introduction
•Line 46: Write it as a complete sentence and put a citation on it. Done
•Provide, a highlight on the various mental illnesses in the region. To the best of our knowledge, there were no prevalence studies done on mental illness in the Kasai region. Therefore, the section from line 68 until 82 discuss the risk factors for poor mental health in the area, and highlight the lack of data.
•Elaborate more on how conflict can lead to mental illnesses with examples. Further details have been added to sentences 51-61.
•Besides, conflict; are there other risk factors that that would exacerbate mental illness in the region that need to be highlighted. These risk factors have now been highlighted as follows: Although it has largely subsided, the conflict has exacerbated risk factors for poor mental health in the area: it left pockets of instability and widespread destruction and destitution, with schools and health centres destroyed. It has worsened pre-existing food insecurity and livelihood crises. In addition, there is said to be a profound gender crisis in most parts of Kasai (9, 10).

4. Methods section
•Line 89: should be Methodology instead of Methods - changed
•Line 90: Design and sample should be separated and should be written as “ Study design” This has now been changed
•In this sub-section, ensure these sections are clear in the Methodology. These sections have all been described
oStudy design
oStudy setting
oStudy population
oSample size
oSelection criteria -both inclusion and exclusion-criteria
oStudy participant recruitment or enrolment
oTool used
oStudy procedures -
oData management and quality control
oStatistical data analysis-
oEthical consideration -issue of consent forms and a statement on Helsinki declaration should be included. Helsinki declaration statement has been added
5. Results
•Line 149: The average age was around 40 years in both samples; Was it net 40 years or 40.---± -- years? This has now been rewritten as follows for clarity: “The average age was 40 years Wave 1 and 42 years in Wave 2”
•Line 150: Should be written as “Demographic characteristics of the study participants and mental health outcome scores” The table has been split and table titles have been updated.
•Though an independent table on detailed “Demographic characteristics of the study participants” should be provided An independent table has been provided (table 2)
•Table 1: The figures should quoted as % (n) other than n (%) as the percentage is the main measure and not frequencies. The table has been updated, neither are now in parenthesis.
•The frequencies such as Formal employment do not add up to the total 200 study participants and please check the figures all through the tables or account for the missing participants. Thank you. Missing data has now been added.
•Table 1 should be converted to Scientific table. I believe the table is in the right format as requested by the journal, but I will email to double check. <https://journals.plos.org/globalpublichealth/s/tables>
•Report overall mean as: Mean±--- other than Mean (SD) and this should be done all through the tables. We have followed APA guidelines, which suggest that Mean and standard deviations are used.
•Line 152: What is the study characteristic? This referred to participant characteristics (so sex, age, and formal employment.)
•In table 2: The values in the rows with brackets (); that do they mean? These are standard deviations, a description has been added at the bottom of the table to clarify.
•Where are the p-values and confidence intervals? Only the mean and standard deviation of quality of life scores were presented, no further comparative analysis was done as these were used in the broader regression model. Therefore p-values and confidence intervals were not calculated/reported at this stage.

6. Discussion
•Should be sub-sectioned according the results sub-sections or domains such as:
oDemographic characteristics of the study participants
oDemographic characteristics of the study participants and mental health outcome scores
oQuality of Life scores
oMultivariable linear regression models for depression and anxiety. The discussion has touched upon all these points, however we feel that it would decrease readability and flow of the discussion if these were discussed as part of separate sections. We’d therefore like to request to keep the current discussion, which we believe has been written in the appropriate format.
•Line 204: Provide citation for the reported other study. This has now been added
•Percentages should be written to one (1) decimal place and all through the document
•What were the limitations of the study? There are several limitations mentioned in the discussion: 1.) we did not measure PTSD. 2) we did not capture experiences of gender inequities. 3) we did not include children and adolescents. 4) this study was not a longitudinal study that collected data from the same participants over time.

Conclusion
•Let the conclusion be drawn on all the 3 domains of the study. A conclusion related to the Quality of Life measurements of the study has now been added.

**Reviewer #4:** Thank you for correcting and reviewing the recommendations previously made by other reviewers. This article is good as it is, but it could be better.

Format comments:
1. Add reference in line 25. A reference to support this statement has been added to line 47 in the introduction.
2. Correct Organisation to Organizartion in line 59. This has been changed
3. Correct "data was" instead of "data were" in line 100. This has been changed.
4. Line 147, it isn't clear if the correct term is "both surveys" or "both waves", please review. This sentence has been re-written as follows for clarity: Table 1 shows the sample characteristics of study participants. These were similar across both waves; with a marginally higher proportion of men and slightly more people who were not in formal employment (e.g. unemployed or subsistence farmers).
5. In line 229 add "on" before PTSD. Thank you, this has been added.

Content comments:
1. It isn't clear, and I think it's an important variable, if the group mental health session was held before or after the survey applications. If it was before, it is important to note as this might have helped participants to better answer the surveys, and this is important for discussion. If it happened after the surveys, this could help explain why the prevalence rates reduced after time? Thank you for raising this important point. The mental health session took place after the data collection was completed. This has been clarified in the methodology. However, since this session was more of a mental health awareness session, and not in-depth counselling or support, we do not expect it would have impacted prevalence rates a year later.

2. If there were literacy issues with the participants, were the surveys administered with someone else and they had to give out the answer? Please clarify and state this clearly. This is important as well because if this was done with the help of someone else, the answers could have been influenced by the need of the participants to not appear so negative and give out negative responses (specially after 1 year). The survey was administered verbally to all participants. This has been added as something to consider in the discussion.

3. Please give further explanation as why you think the prevalence rates reduced on wave 2, this is such an interesting finding!!! Thank you! Whilst univariable analysis appeared to show a reduction, our multivariable analysis showed that the passing of time no longer significantly predicted a reduction in prevalence rates, when accounting for differences in gender, age and quality of life. Environmental quality of life, however, appeared to influence women’s mental health more than men’s mental health. We have now added a hypothesis that increased feelings of safety might have explained the reduction in anxiety levels for women.

Good luck!

**Reviewer #5:** Dear Authors, this paper is well written and pertains a vital part of mental health in conflict area DRC which would affect the livelihood of the population. There are some minor revisions that need to be corrected before publication. Thank you!

- Background; you should integrate other studies done within the East African region like Northern Uganda and South Sudan or even Burundi with the recent conflicts. There have also been studies done to assess the mental health of refugees from the DRC in Uganda. that would help to add weight and relevance to the paper. Thank you. We have added some further information and references on the prevalence rates of PTSD, depression and anxiety in East Africa (including studies from DRC, Uganda, Rwanda, S-Sudan)
- There have also been studies that suggest the mental health interventions that have been made within the country, Could you also highlight those and the interventions in place and if they could affect your paper. We found no papers about mental health interventions in the Kasai region that we could use here – we would be grateful if the reviewer could direct us to these studies. We have added reference to two interventions that provide socio-economic support with good results.
- Discussion; the discussion states that time was not a significant aspect in the PHQ and anxiety scale scores but were there some interventions by either local government or organizations during the time period in the study areas that need to be noted or the status of the community remained the same within the one year time period that could have affected your results. Thank you very much for this important point. To the best of our knowledge, there have been no targeted interventions in the area. This has been added to the methodology.
